# Factors contributing to post-stroke health care utilization and costs, secondary results from the life after stroke (LAST) study

**DOI:** 10.1186/s12913-020-05158-w

**Published:** 2020-04-06

**Authors:** Øystein Døhl, Vidar Halsteinli, Torunn Askim, Mari Gunnes, Hege Ihle-Hansen, Bent Indredavik, Birgitta Langhammer, Ailan Phan, Jon Magnussen

**Affiliations:** 1 Department of Public Health and Nursing, Faculty of Medicine, Norwegian University of Science and Technology, P.O. Box 8905 MTFS, N-7491 Trondheim, Norway; 2 Department of Health and Social Services, City of Trondheim, Norway; 3 St. Olavs University Hospital, Trondheim, Norway; 4 Department of Neuromedicine and Movement Science, Faculty of Medicine, Norwegian University of Science and Technology, Trondheim, Norway; 5 Department of Medicine, Vestre Viken, Bærum Hospital, Sandvika, Norway; 6 Department of Neurology, Oslo University Hospital, Oslo, Norway; 7 Stroke Unit, Department of Medicine, St. Olavs Hospital, Trondheim, Norway; 8 Faculty of Health Sciences, Oslo Metropolitian University, Oslo, Norway; 9 Sunnaas HF, Nesodden, Norway

**Keywords:** Stroke, Economics, Cost, Health care utilisation, Quality of life

## Abstract

**Background:**

The result from the Life After Stroke (LAST) study showed that an 18-month follow up program as part of the primary health care, did not improve maintenance of motor function for stroke survivors. In this study we evaluated whether the follow-up program could lead to a reduction in the use of health care compared to standard care. Furthermore, we analyse to what extent differences in health care costs for stroke patients could be explained by individual need factors (such as physical disability, cognitive impairment, age, gender and marital status), and we tested whether a generic health related quality of life (HRQoL) is able to predict the utilisation of health care services for patients post-stroke as well as more disease specific indexes.

**Methods:**

The Last study was a multicentre, pragmatic, single-blinded, randomized controlled trial. Adults (age ≥ 18 years) with first-ever or recurrent stroke, community dwelling, with modified Rankin Scale < 5. The study included 380 persons recruited 10 to 16 weeks post-stroke, randomly assigned to individualized coaching for 18 months (*n* = 186) or standard care (*n* = 194). Individual need was measured by the Motor assessment scale (MAS), Barthel Index, Hospital Anxiety and Depression Scale (HADS), modified Rankin Scale (mRS) and Gait speed. HRQoL was measured by EQ-5D-5 L. Health care costs were estimated for each person based on individual information of health care use. Multivariate regression analysis was used to analyse cost differences between the groups and the relationship between individual costs and determinants of health care utilisation.

**Results:**

There were higher total costs in the intervention group. MAS, Gait speed, HADS and mRS were significant identifiers of costs post-stroke, as was EQ-5D-5 L.

**Conclusion:**

Long term, regular individualized coaching did not reduce health care costs compared to standard care. We found that MAS, Gait speed, HADS and mRS were significant predictors for future health care use. The generic EQ-5D-5 L performed equally well as the more detailed battery of outcome measures, suggesting that HRQoL measures may be a simple and efficient way of identifying patients in need of health care after stroke and targeting groups for interventions.

**Trial registration:**

https://www.clinicaltrials.govNCT01467206. The trial was retrospectively registered after the first 6 participants were included.

## Background

Stroke is a major contribution to loss of health as well as a cause of death. Globally, stroke is the second most frequent cause of death with nearly 6 million deaths worldwide in 2016 [1]. However, improvements in stroke care means that most patients now experience significant improvement in function during the first weeks and months after stroke. Functional level three to six months post-stroke is strongly associated with long-term outcome [2, 3]. There is, however, a lack of evidence concerning effective interventions to prevent functional decline in the years after stroke. In the Life After Stroke study (the LAST-study) the efficacy and safety of an 18-month follow-up programme of individualized regular coaching on physical activity and exercise was evaluated [4]. Results from the LAST-study indicated that: “Regular individualized coaching did not improve maintenance of motor function, nor secondary outcomes, compared to standard care” [4]. Even though this study did not find any significant differences in the primary outcomes, it would still be of interest to examine potential differences of utilization of health care services. The purpose of this paper was to analyse the use and associated cost of health care services, with a specific focus on primary care services.

We addressed three research questions: Despite the fact that the intervention did not significantly improve outcome, a potential reduction in the use of health care services may still mean that the LAST intervention could reduce costs in the long term perspective. Thus, taking a health care perspective we first tested for differences in total health care costs between the intervention and control group.

Second, we analysed to what extent individual differences in health care costs could be explained by individual and environmental factors such as place of residence [5, 6].

Clinical studies regularly use clinical endpoints that are specific or considered as relevant to the particular patient population studied. Economic evaluations, on the other hand, usually prefer generic outcome measures such as preference based measures of health related quality of life (HRQoL). Although the LAST-study found no improvement in the HRQoL secondary outcome measure, the third issue raised in this paper is whether a generic HRQoL measure perform better or worse than clinical endpoints in predicting the use of health care services for patients with stroke.

To analyse these issues we utilized a framework originally proposed by Anderson & Newman [7, 8]. This framework is frequently used to identify and structure individual determinants of health care use. Three types of variables believed to explain the need for health care services are described. *Predisposing factors* are socio-cultural characteristics of individuals that exist before the onset of illness, *enabling factors* can be described as the logistics of obtaining care and *need factors* are characteristics related to the (perceived) health of the individual.

Need variables like increased physical and cognitive disability are strong predictors on the use of primary care services [6, 9–14]. Even though increased disability is the most important indicator for the use of health care services also predisposing factors such as age and gender may also explain the use of primary care services. Age is reported to be a strong predictor of primary care [6, 10, 13, 15, 16]. The effect of gender is less conclusive [10, 15, 17]. Living arrangements and access to informal care represent potential enabling factors. People who live alone have higher use of formal care than people who live with spouses [10, 12, 15, 18].

## Methods

LAST [4] was a multicentre, single-blinded, pragmatic, randomized controlled trial (RCT) performed at two hospitals in Norway: Trondheim University Hospital and Bærum Hospital. The trial was performed together with the primary healthcare service in the municipalities of Trondheim, Asker, and Bærum. Those included were adults, above 18 years old, living at home, with first-ever or recurrent stroke, no serious comorbidities and with modified Rankin Scale (mRS) less than five. Patients were enrolled at the outpatient clinic 10–16 weeks after onset of stroke. The patients were randomly assigned. The randomization was in blocks of two and four. The intervention group received regular individualized coaching on physical activity for 18 months, while the control group received standard care [4]. It has been shown that the participants established and maintained moderate-to-good adherence to the intervention [4, 19]. Standard care received by the control group usually consisted of less than 1 h physiotherapy per week, often limited to the first 3 months for patients with mild to moderate strokes but could last for up to 6 months for patients with the most severe strokes and for selected patients even longer. Primary outcome was the Motor assessment scale (MAS), measured at the end of the follow-up period. Secondary outcomes were mRS, Berg balance scale (BBS), Barthel index (BI), Gait speed, Six minute walk test, Timed up and go test and Stroke impact scale (SIS). HRQoL was registered using the EQ-5D-5 L questionnaire. The different health states generated by EQ-5D-5 L was assigned values from the UK tariff when calculating the EQ-5D index [20]. Table 1 shows baseline characteristics used in this study of the patient population [4].

**Table 1:** Baseline demographic and clinical characteristics

	Intervention group (*n* = 186)	Control group (*n* = 194)
Predisposing variables		
Age (years), mean (SD)		
y, mean (SD)	71.7 (11.9)	72.3 (11.3)
Age		
≥ 80 – n (%)	44 (23.7)	53 (27.3)
Gender		
Female – n (%)	82 (44.1)	67 (34.5)
Enabling variables		
Living alone – n (%)	56 (30.1)	51 (26.3)
Need variables		
MAS, mean (SD)^a^	41.8 (6.9)	41.7 (7.4)
Gait speed mean (SD)^a^	1.29 (0.55)	1.36 (0.60)
MMSE, mean (SD)^a^	27.9 (2.32)	28.0 (2.30)
HADS, mean (SD)^a^	6.6 (5.3)	7.2 (6.1)
Barthel, mean (SD)^a^	96.3 (7.4)	96.1 (9.2)
mRS, mean (SD)	1.45 (1.08)	1.44 (1.10)
EQ-5D-5 L, mean (SD)^a^	0.83 (0.16)	0.83 (0.17)
Costs		
Grand total, mean (SD)	23,126 (30780)	20,412 (32114)
Grand total ex intervention, mean (SD)	21,646 (32114)	
Hospital, mean (SD)	9453 (16936)	9201 (13199)
*Out-patients’, mean (SD)*	*2325 (3430)*	*1796 (1829)*
*In-patients’ day, mean (SD)*	*203 (609)*	*214 (866)*
*In-patients’, mean (SD)*	*6925 (15413)*	*7191 (12814)*
Primary care, mean (SD)	9551 (20748)	8491 (23792)
*Home care, mean (SD)*	*6117 (15101)*	*6054 (18222)*
*Nursing home, mean (SD)*	*3434 (12270)*	*2437 (10970)*
Physiotherapists, mean (SD)	3169 (3366)	1667 (3412)
Physiotherapists ex intervention, mean (SD)	1689 (3166)	
GP’s, mean (SD)	953 (847)	1053 (947)

^a^Results from the pooled data. *SD* Standard Deviation, *MAS* Motor Assessment Scale, *MMSE* Mini-Mental State Examination, *HADS* Hospital Anxiety and Depression Scale (HADS A and D), *mRS* modified Rankin Scale; modified Rankin Scale (mRS), Health Related Quality of Life (EQ-5D-5 L)

The estimation of the sample size, which was based on the primary outcome of the main study (Motor Assessment Scale at 18 month follow-up), has been reported elsewhere [4]. Research assistants screened patients for eligibility and did all assessments face-to-face at inclusion and at follow-up. The assistants were blinded to allocation of the treatment. Randomization of the patients was performed by a system developed and administered by the Unit of Applied Clinical Research, Faculty of Medicine, Norwegian University of Science and Technology, Trondheim, Norway [4].

In Norway health care services is divided into specialised care and primary health care, both are a part of the welfare system. Health care services are provided within a public and tax based health care system. The responsibility for primary health care is devolved to municipalities. Primary care constitutes of long term care (LTC), general practitioner (GP) and physiotherapists. LTC may be provided both in an institution or at home. The municipalities will both operate and finance primary health care services, with some financial contribution from recipients.

Type of health care services and their associated unit costs are shown in the Supplemental material. We differentiated between general practitioner (GP) services, physiotherapy services (private and public), primary care services (mainly home health care and rehabilitation/nursing homes) and hospital care. Information about GP services and private physiotherapy services was retrieved from the Norwegian health economics administration (HELFO). Use of public physiotherapy services, home health care and rehabilitation/nursing homes were provided by the participating municipalities. Use of specialized health care (hospital inpatient, day-care and outpatient) was obtained from the Norwegian patient registry. Most of the home care services were measured in hours per week (cf. Supplemental material), while institutional care was measured in number of days. For patients from the municipality of Bærum it was not possible to separate the number of hours for the intervention on an individual level. As a proxy for the intervention cost per patient in Bærum we therefore used the average intervention costs from patients in Trondheim and Asker. For each type of health care there is an associated unit cost. Unit cost of GP’s and private physiotherapy services was provided by HELFO, unit cost of primary and hospital care was based on cost information from the municipality of Trondheim and St. Olav hospital, respectively. Indirect costs as e.g. travel expenses were not included. All costs are in Norwegian kroner, but is presented in Euros, using an exchange rate of 9.58 NOK/Euro, which is based on the monthly average exchange rate January to July 2018 [21].

### Statistical analysis

Differences between the control and intervention and the relationship between health care costs and predisposing, enabling and need factors was analysed using multivariate regression analysis. We did separate analyses for the costs of the municipality-, hospital-, GP services in addition to the aggregated grand total costs. Because of a skewed distribution of the error term, the dependent variable was transformed into natural logarithm, and a 2-stage model was used to correct for bias in the dataset [22].

1 $$ \ln {y}_{ij}=\gamma +{\sum}_{m=1}^M{\theta}_{mj}{x}_{mi}+{\sum}_{l=1}^L{\beta}_l{x}_{li}+\delta {x}_{ti}={r}_i $$

Where:

ln*y*_*ij*_ – Individual cost for a person i for service j, measured as logarithm.

γ_j_ – The grand mean of ln*y*_*ij*_.

*x*_*m*_– A set of M individual need variables.

*x*_*l*_ – A set of L other predisposing and enabling variables.

*x*_*t*_ – A dummy variable measuring whether person i is randomised to intervention or not.

r_i_ – Individual error term assumed to be normally distributed with constant variance.

For a continuous variable, the estimated value ${\hat{\theta}}_m$ has an interpretation as percentage increase in y (cost) with one unit increase in x. The interpretation of the categorical (“dummy”) variables is percentage difference between the two groups. For categorical variables we used Kennedy’s approximation to adjust for bias [23, 24].

The *predisposing* variables included in the individual analysis were *age* and *gender.* Age could affect both the risk of stroke and the effect of medical treatment [25, 26]. In this study we investigated whether age and gender were related to cost differences after controlling for disability.

The *enabling* variables included were whether the individual was living alone (*cohabitation).* We also included dummy variables for the control group and for the resident municipality of the individuals.

Finally, we used two different specifications of need variables measured at baseline, 10–16 weeks post stroke: In the first, the *need* variables were a selection of the outcome variables from the effect study, representing the domains of body functions (e.g. motor function and cognitive function) and activities and participation (e.g. mobility, ADL function and dependency) according to ICF [27]. The primary outcome MAS was developed for persons with stroke to assess motor function [28]. The Barthel index [29] and the mRS to assess independence of activities of daily living [30]. Other measures included were 10-m maximum Gait speed to assess mobility, the Mini Mental State Examination (MMSE) to assess cognitive function and the sum of HADS A and HADS D to measure anxiety and depression [31–33].

In the next specification we included only a generic measure of HRQoL using EQ-5D-5 L measured at baseline. Thus, in this setting we did not calculate the effect of the intervention in terms of QALYs gained, but rather use the EQ-5D index as a measure of HRQoL post stroke.

For health care services (and thereby costs), predisposing and enabling variables there were no missing data. For the need variables there were some missing data. Twenty-seven patients had a least one missing need observation, 11 were in the intervention group and 16 in the control group. There were no missing mRS observations and only one BI and two MAS observations. However, there were 19 missing Gait speed observations. For the MMSE there were four missing and five for HADS A and D (patients missing on A were also missing on D). The EQ-5D-5 L had 10 missing values. Data were imputed using a conditional regression imputation with 100 imputations and up to 100 iterations for each imputation [34, 35]. Predisposing, enabling and need variables were used in the imputation.

The LAST study was conducted in accordance with the institutional guidelines and was approved by the Regional Committee of Medical and Health Research Ethics (REC no. 2011/1427). Due to Norwegian regulations and conditions for informed consent, the dataset will not be publicly available before it is anonymized at earliest in 2025. The study was registered with Clinicaltrials.gov (NCT01467206). Complete details of this study protocol have been published elsewhere [36].

The LAST study follows the CONSORT guidelines. All analyses were done using IBM statistics SPSS version 25 and Stata version 15.1.

### Patient and public involvement

A person from the patient organization took part in the steering committee and participated in stages of the project from writing the protocol until publication. The research questions were discussed with the patients, they were however not involved in the design of the study or the recruitment to the study. The burden of the intervention was discussed in the meetings with the patients. Further will the results from the study be presented in the “Slagordet”, a publication from the Norwegian association for stroke affected.

## Results

From Table 1 we see that the average grand total cost were 23,126 Euro in the intervention group, of this in average 1480 Euro were costs related to the intervention. In the control group the average grand total cost were 20,412 Euro. The hospital costs constituted nearly 41% of the total costs in the intervention group and 45% in the control group. The primary care costs constituted 41% of the total costs in the intervention group and 42% in the control group. The higher cost of physiotherapy in the intervention group is due to the cost of the intervention. In intervention group there were a smaller proportion of elderly above 80 years, but there were a higher proportion of females and those living alone.

Tables 2 and 3 shows the results from the two regression models. There were higher total health care costs in the intervention group than the control group, related to the intervention cost. For the primary care and hospital care there were no cost differences between the intervention and control group. For the GP’s there were indication (*p* = 0.09) of lower costs in the intervention group.

**Table 2:** Regression results^a^ of individual cost coefficient measured as proportion increase in cost with one unit increase, with 95% CI, *n* = 380

	Total costs	Primary care	Hospital care	GP
**Intervention**	0.31† (0.05–0.64)	0.47 (− 0.16–1.58)	− 0.08 (− 0.32–0.24)	−0.22 (− 0.42–0.03)
**Need variables**				
MAS	−0.04† (− 0.07 − − 0.01)	−0.05 (− 0.11–0.01)	−0.02 (− 0.06–0.02)	0.01 (− 0.03–0.04)
Gait speed	−0.41† (− 0.63 − − 0.18)	−0.68† (− 1.32 − − 0.05)	−0.25 (− 0.55–0.05)	−0.29† (− 0.57 − − 0.00)
MMSE	−0.04 (− 0.09–0.01)	−0.17† (− 0.29 − − 0.05)	−0.04 (− 0.10–0.03)	−0.04 (− 0.10–0.02)
HADS	0.03† (0.00–0.05)	0.01 (− 0.04–0.07)	0.02 (−0.01–0.05)	0.04† (0.01–0.06)
Barthel	−0.003 (− 0.02–0.02)	−0.01 (− 0.06–0.03)	0.02 (− 0.01–0.04)	0.01 (−0.02–0.04)
mRS	0.27† (0.12–0.43)	0.18 (− 0.19–0.55)	0.18 (−0.03–0.39)	0.23† (0.02–0.43)
**Predisposing variables**				
Age 60–69	0.14 (−0.22–0.66)	−0.68 (− 0.91–0.22)	0.35 (− 0.18–1.24)	0.17 (−0.28–0.90)
Age 70–79	0.15 (− 0.19–0.65)	−0.48 (− 0.85–0.79)	0.22 (− 0.24–0.96)	0.50 (−0.05–1.36)
Age 80–89	0.40 (− 0.05–1.08)	0.06 (− 0.68–2.55)	0.16 (−0.31–0.96)	0.45 (− 0.12–1.40)
Age 90+	0.40 (− 0.32–1.86)	−0.33 (− 0.87–2.32)	−0.18 (− 0.69–1.16)	−0.22 (− 0.69–0.93)
Gender	−0.02 (− 0.24–0.26)	−0.32 (− 0.63–0.24)	−0.06 (− 0.33–0.33)	0.03 (− 0.25–0.42)
**Enabling variables**				
Living alone	0.35† (0.03–0.77)	0.83† (0.00–2.37)	0.30 (− 0.09–0.87)	−0.15 (− 0.40–0.19)
**Other variables**				
Asker	0.66† (0.19–1.32)	1.49† (0.06–4.82)	0.40 (− 0.10–1.18)	0.22 (− 0.21–0.86)
Bærum	0.71† (0.31–1.25)	2.81† (0.94–6.45)	0.10 (−0.24–0.58)	0.29 (− 0.09–0.83)
Constant	13.70† (11.40–16.01)	18.19† (13.08–23.29)	10.50† (7.47–13.52)	8.48 (5.54–11.42)
Adj. R-square	36.7	32,3	4.5	7.8

^a^Results from the regression †*P* < 0.05

**Table 3:** Regression results^a^ of individual cost coefficient measured as proportion increase in cost with one unit increase, with 95% CI, *n* = 380

	Total costs	Primary care	Hospital care	GP
**Intervention**	0.35† (0.06–0.71)	0.44 (− 0.22–1.67)	−0.07 (− 0.31–0.26)	−0.21 (− 0.41–0.05)
**Need variables**				
EQ-5D-5 L	−3.50† (− 4.26 − − 2.75)	−3.48† (− 5.22 − − 1.74)	−2.19† (− 3.14- -1.25)	−2.15 (− 3.05 − − 1.25)
Predisposing variables				
Age 60–69	0.42 (− 0.05–1.12)	−0.72 (− 0.94–0.23)	0.60 (− 0.04–1.66)	0.29 (−0.21–1.09)
Age 70–79	0.47† (0.01–1.14)	−0.51 (− 0,87–0.93)	0.37 (− 0.15–1.20)	0.63† (0.04–1.56)
Age 80–89	1.23 (0.48–2.36)	0.31 (−0.67–4.14)	0.45 (− 0.14–1.43)	0.71† (0.05–1.79)
Age 90+	0.92 (−0.11–3.15)	−0.30 (− 0.89–3.51)	−0.07 (− 0.66–1.53)	−0.14 (− 0.65–1.14)
Gender	0.06 (− 0.19–0.38)	− 0.29 (− 0.63–0.39)	−0.05 (− 0.32–0.34)	0.11 (− 0.19–0.52)
Enabling variables				
Living alone	0.35† (0.01–0.80)	0.63 (− 0.17–2.22)	0.30 (−0.10–0.87)	−0.17 (− 0.41–0.17)
Other variables				
Asker	0.50† (0.07–1.12)	1.36† (0.00–5.06)	0.30 (− 0.16–1.03)	0.27 (− 0.16–0.91)
Bærum	0.60† (0.23–1.09)	2.60† (0.81–6.16)	0.05 (−0.25–0.47)	0.33 (− 0.03–0.83)
Constant	13.39† (12.66–14.12)	12.47† (10.66–14.28)	11.87† (10.95–12.79)	10.09† (9.22–10.96)
Adj. R-square^1^	28.2	19.3	6.3	7.1

^a^Results from the regression †*P* < 0.05

In the specification using clinical outcome measures, MAS and Gait speed were both strongly associated with the use of health care services, whereas there was no such association for Barthel. MMSE was associated with the use of primary care costs. Gait speed, on the other hand was negatively associated with all types of health care services, except hospital care. Implying that increased Gait speed reduced the use of health care services, and thereby costs.

*Age and gender* were not associated with primary health care costs in any of the model specifications.

There was a positive association between living alone and the use of primary care and total costs.

Substituting the condition specific outcome measures with the generic EQ-5D index did not substantially change the measures of association between age, gender or living conditions and the use of health care services. The negative association between EQ-5D index and the use of health care was also statistically significant for all types of health care.

Patients from Asker and Bærum had higher health care costs than patients from Trondheim. This was mainly due to higher primary care costs.

The models were also estimated with interaction terms between explanatory variables and the intervention variable (results not shown). None of the interaction terms were statistically significant.

**Fig. 1:**
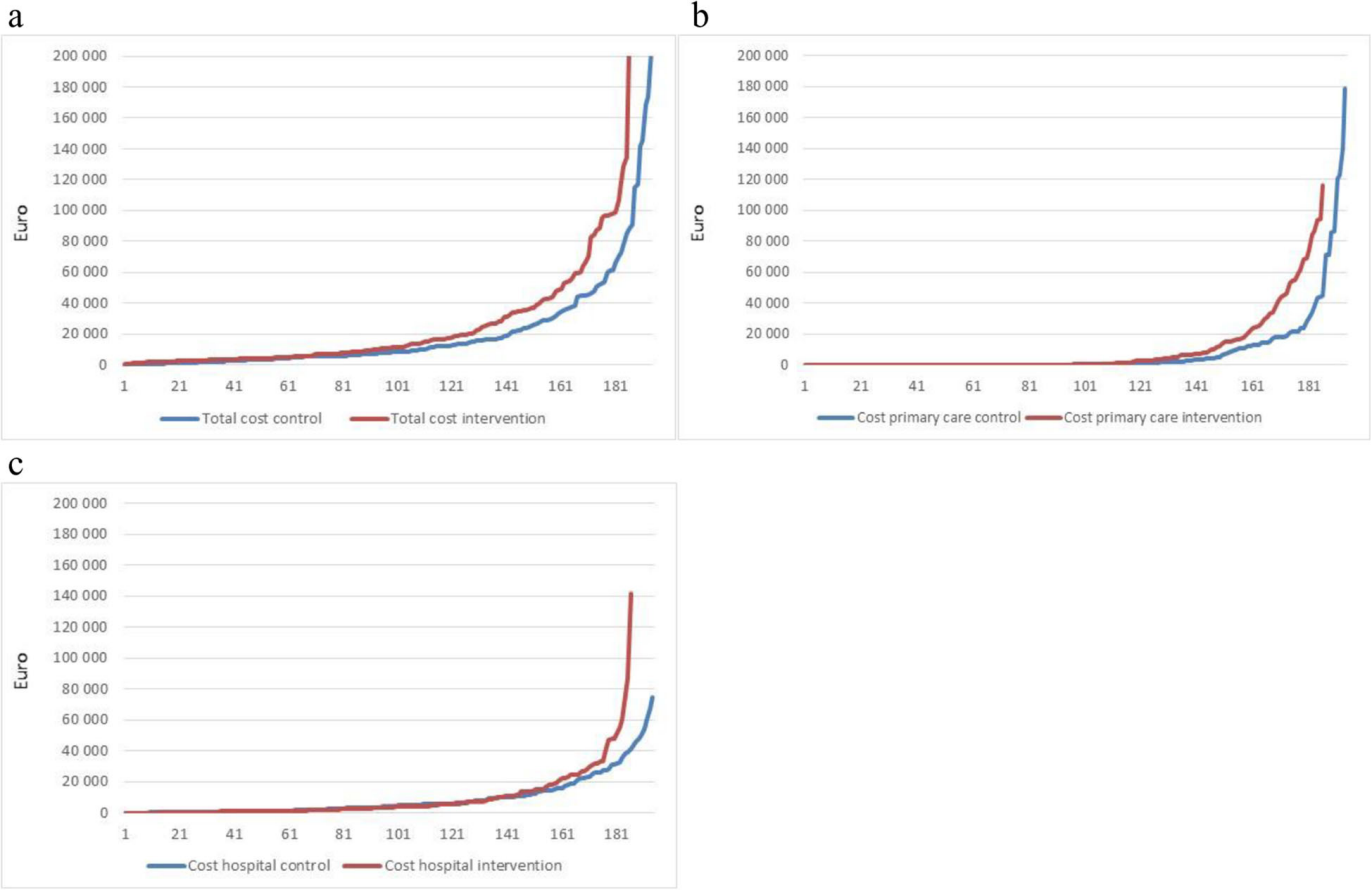
Patients sorted due to individual cost. **a** Increasing total cost per patient measured in Euro, average = 21,741€; **b** Increasing primary cost per patient measured in Euro, average = 9010€; **c** Increasing hospital cost per patient measured in Euro, average = 9324€

The use of health care services was unevenly distributed among the study participants. Figure 1 shows the individual cost per patient, sorted from lowest to highest cost for the control and intervention group. Figure 1a) shows the total cost for all services. The individual cost during the 18-month period ranged from 11 Euro to 204.6 thousand Euro, with an average of 21.7 thousand. In the case of primary care services 195 (51.3%) out of 380 of the study participants did not receive any services at all (Fig. 1b), and 19% of the participants generated 90% of total costs in primary care. The share of patients using hospital services was somewhat higher (358 of 380) (Fig. 1c). In this case 42.6% of the participants generated 90% of the total costs.

## Discussion

Results from the present study showed that the LAST intervention did not led to a reduction in the health care utilisation compared to standard care. The individual coaching did imply increased physiotherapy costs compared to standard care. Similar costs were the case for primary care costs and hospital costs. There were slightly lower GP costs in the intervention group, this could be related to somewhat higher use of out-patient hospital care. Our findings were in line with results from a home-based intervention study from Denmark. In this study they found that the intervention group achieved better mRS, but the cost savings were outweighed by increased intervention costs [37]. Earlier studies have found that physical and cognitive impairment are crucial determinants of primary health care use [5, 6, 14, 38]. The result from the Life After Stroke (LAST) study showed that the follow up program, did not improve maintenance of physical or cognitive functioning. Therefore, our findings are not surprising. However, a potential obstacle in our analysis could be the relatively high share of who were capable of carrying out all usual activities. Consequently, a high number of the patients had low or nearly no costs and a relatively large share (around 14%) of the patients had health care costs below the estimated cost of the intervention. A high share of independent and low cost patients can potentially hide potential benefits for more disabled patients from the follow-up program. Further research should investigate whether a more customized follow-up program could be effective and whether an intervention that is more targeted at patients with a high probability of health care use could reduce costs.

There were differences in the use of health care between the three municipalities included in this study. These differences were related to the use of hospitals services, in which the two neighboring municipalities of Asker and Bærum had substantially higher costs than those from Trondheim. We do not have enough information to fully explain this, but patients from Asker and Bærum were generally admitted to a local hospital, while patients from Trondheim were admitted to a highly specialised university hospital. Thus, both differences in admission policies and capacity may partly explain the differences in costs. One implication of this finding could be that the amount of care patients receives depend on where they are living. Remembering that these findings were within a public health care system where equity is a central goal, this result should be a subject for further research.

Of the two predisposing variables included we did not find age or gender to be a strong determinant for use of primary health care services. A positive association between age and the use of home health care has been found in other studies [5, 6, 15]. The lack of association between age and the use of hospitals is somewhat surprising. Neither the use of GPs nor the physicians were related to age. One explanation could be that increasing use of nursing homes among the old also imply that they will get both GP and physician services as an integrated part of the nursing home stay. Thus, the lack of association between increased age and the use of GPs was not surprising. After controlling for disability, we did not find any gender related differences in the use of health care services. Previous studies indicate that older women are at greater risk of stroke, increasing disability and higher risk to be institutionalized after stroke [39–41]. One possible reason why this study did not find any gender related differences could be that higher use of institution was substituted by higher use of other health care services as hospital care or home care.

The enabling variable, living status, was positively associated with the use of primary care services. Thus, individuals living alone were likely to use more primary care services than those living with a cohabitant. For the general population of home care users in one of the municipalities included in this study (Trondheim) it has been shown that female users living with men received significantly more help than male users living with women [5]. For stroke rehabilitation patients this association could, however not be established.

Most of the clinical need factors were, as expected, strongly associated with the use of health care services. When both MAS and Barthel Index were included, only the first was significantly associated with health care use. We also note that the cognitive functioning of the patients, as measured by MMSE, was only associated with primary health care use. Also other studies have found that nursing home admission and home care use increase with increased cognitive disability for elderly persons [5, 6, 42]. Again, a relatively high proportion of the patients were well functioning and were not, yet, users of long-term services from the municipalities.

On the other hand, Gait speed was negatively associated with all forms of health care use. Thus, this simple test may provide valuable information about the possible need for primary care, hospital care, GP visits and physiotherapy. Gait speed as a predictor for functional decline is found in other studies [43, 44]. We did not find any significant effect of the Barthel index. The Barthel index constitutes of only ADL and mobility variables, ADL variables might have a ceiling effect, making it difficult to detect differences among elderly with low disability [45]. So a possible reason for this could be that gait speed is sensitive in detecting differences among stroke survivors with higher ability.

Replacing the condition-specific need variables with the generic EQ-5D index did not change the estimates of the other variables in the model. However, EQ-5D index was negatively associated with all types of health care use, and in particular, the association between baseline EQ-5D index and the use of primary care services was strong. While the clinical endpoints used in our first model altogether explained a higher share of health care costs, the EQ-5D index performed almost as well and seems to be a better predictor of different types of health care costs. Using EQ-5D index also facilitates comparisons between interventions aimed at different types of patients. Indicating that a simple index as EQ-5D could predict costs as well as more detailed indexes.

The main strength of this project was the randomised controlled study design including a rigorous recording of health care data from several different sources. The 18-month follow-up period was also unique, making it possible to estimate the total costs related to stroke care in the long term. Furthermore, the large sample size and very good completeness of data should also be regarded as a strength. However, there were also some limitations to this approach. First, the analysis was limited to public funded costs, thereby leaving out the amount of care given by cohabitants or other relatives. Earlier results indicate that cohabitants can act as a substitute for public care [5, 15]. Second, the comparability of data across municipalities was somewhat reduced as Asker and Trondheim registered actual face to face time in their electronic patients record, while the numbers from Bærum reflected the administratively allocated time to each patient.

After controlling for other differences there seem to be relatively large differences in received care whether the patient where discharged from Bærum hospital or St. Olavs hospital. Further analysis (not shown her) indicates that these differences were related to inpatient and outpatient care, but not day-care. These differences were not reflected in other services like GP services. This result indicates that patients from Bærum and Asker has 50–60% higher costs than those from Trondheim. Further research should study whether differences in hospital care is reflected in better outcome for the patients.

According to the annual report from the Norwegian stroke registry, about 60% of the Norwegian stroke population in 2018 had a mRS score of 2 points or better at 3 months follow-up compared to 79% in the LAST-population at the same time point [46]. In more detail, the proportion categorized with mRS = 0 and mRS = 2 were comparable in both groups, while a greater proportion was categorized with mRS = 1 in the LAST- population (41% versus 21%). This could imply that the average costs (Table 1) is a bit higher in the overall stroke population. But this does not necessarily affect the estimated effects from Table 2.

## Conclusion

We found, in line with the previously reported primary outcome from this study, that an 18-month regular individualized coaching program did not reduce health care costs compared to standard care. However, the skewed distribution of costs among patients suggests that an intervention targeted at likely high cost patients could yield different results. Using the Anderson-Newman framework we found predictors for future health care use both among need factors, enabling factors and predisposing factors. However, a generic measure of health related quality of life (EQ-5D-5 L), performed equally well in predicting the individual use of resources as the more traditional battery of clinical outcome measures. This suggests the HrQoL measures may be a simple and efficient way of identifying patients in need of health care after stroke, as well as targeting groups for future interventions.

## Supplementary information

**Additional file 1.** Table Unit cost 2014, measured in Euro.

## Data Availability

Due to Norwegian regulations and conditions for informed consent, the data set cannot be publicly available before it has been anonymized. This will happen at earliest by the end of 2025. For access to data please contact Torunn Askim, NTNU, Faculty of Medicine and Health Sciences, Department of Neuromedicine and Movement Science, N-7491 Trondheim, Norway or torunn.askim@ntnu.no.

## Abbreviations

LAST: Life After Stroke
HRQol: Health-Related Quality of Life
MAS: Motor Assessment Scale
HADS: Hospital Anxiety and Depression Scale
MRS: Modified Rankin Scale
BI: Barthel Index
MMSE: Mini Mental State Examination
EQ-5D-5 L: Euroqol - five dimension – five level instrument for measuring health status
QALY: Quality-adjusted Life Year
GP: General Practitioner
ADL: Activities of Daily Living
HELFO: Norwegian health economic administration
SD: Standard Deviation
