# Supplementary material for: Factors contributing to post-stroke health care utilization and costs, secondary results from the life after stroke (LAST) study
Source: BMC Health Serv Res. 2020 Apr 6;20:288. doi: 10.1186/s12913-020-05158-w (PMC7137416; doi:10.1186/s12913-020-05158-w)
Supplement: Supplementary file 1 — Additional file 1. Table Unit cost 2014, measured in Euro. [file 12913_2020_5158_MOESM1_ESM.pdf]

Supplemental Material

Table Unit cost 2014, measured in Euro

Services type	Type of unit	Unit cost	Source of information – unit cost
Physiotherapist - municipality	Hours	84	Muncipality of Trondheim
Physiotherapist - private	Visits	*	Helfo
Occupational therapist	Hours	80	Muncipality of Trondheim
Long term stay	Days	251	Muncipality of Trondheim
Short term stay - rehabiliation	Days	320	Muncipality of Trondheim
Short term stay	Days	267	Muncipality of Trondheim
Rehabilitation stay	Days	298	Muncipality of Trondheim
Day based rehabilitation	Days	163	Muncipality of Trondheim
Ambulatory follow-up	Hours	78	Muncipality of Trondheim
Home nursing care	Hours	81	Muncipality of Trondheim
Home care services	Hours	78	Muncipality of Trondheim
Safety alarm	Months	4	Muncipality of Trondheim
Meals on wheels	Months	17	Muncipality of Trondheim
Visits to daycentre	Months	553	Muncipality of Trondheim
General practitioner	Visits	*	Helfo
Hospital stay - somatic ward	Days	1 029	St Olavs hospital - local cost-per-patient system
Hospital day-stay	Days	639	St Olavs hospital - local cost-per-patient system
Outpatient visit - somatic ward	Visits	282	St Olavs hospital - local cost-per-patient system

* Costs were based on national fee-for-service tariffs
